# Mineral Raw Material Supply Chain Transparency and Traceability: Does Provenance Matter in the Supply Chain?

**DOI:** 10.1007/s00501-022-01274-8

**Published:** 2022-09-12

**Authors:** Valentina Dietrich, Frank Melcher

**Affiliations:** Department für Angewandte Geowissenschaften und Geophysik, Lehrstuhl für Geologie und Lagerstättenlehre, Montanuniversität Leoben, Peter-Tunner-Straße 5, 8700 Leoben, Austria

**Keywords:** Fingerprinting, Traceability, Supply chains, Fingerprinting, Rückverfolgbarkeit, Lieferketten

## Abstract

Raw material supply chains are complex systems. They build on the presence of economically mineable mineral commodities that will undergo several steps until they are finally being used as consumer products. Trustworthiness into the transparency of a supply chain is of increasing importance, both to upstream and downstream companies. Any deviation from best-practice and quality standards in mining, processing and production is critically looked at by consumers. Therefore, certification systems and proof of origin concepts have emerged within the past years, aiming at providing transparency to supply chains. The analytical proof of origin for mineral raw materials could be beneficial to certification schemes in several ways. It represents the least corruptible method of provenance analysis as it relates directly to the chemical composition of the raw material. Other methods, such as documents, tracers, or barcodes, can be outmanoeuvred in one way or another.

## Abstract (de)

Rohstofflieferketten sind komplexe Systeme. Sie bauen auf wirtschaftlich abbaubaren mineralischen Rohstoffen auf, die mehrere Schritte durchlaufen, bis sie schließlich als Endprodukte konsumiert werden. Die Vertrauenswürdigkeit und Transparenz einer Lieferkette ist für Unternehmen von zunehmender Bedeutung. Jede Abweichung von Best-Practice- und Qualitätsstandards bei Abbau, Verarbeitung und Produktion wird von den Verbrauchern gerade in der heutigen Zeit kritisch beäugt. Daher sind in den letzten Jahren Zertifizierungssysteme und Konzepte für den Herkunftsnachweis von mineralischen Rohstoffen entstanden, die für Transparenz in den Lieferketten sorgen sollen. Der analytische Herkunftsnachweis von mineralischen Rohstoffen könnte für Zertifizierungssysteme in mehrfacher Hinsicht von Vorteil sein. Er stellt die am wenigsten korrumpierbare und veränderbare Methode von Herkunftsnachweisverfahren dar, da er sich direkt auf die chemische Zusammensetzung des Rohstoffs bezieht. Andere Methoden, wie z. B. Dokumente, Tracer, Barcodes o. Ä., können auf die eine oder andere Weise umgangen werden.

## Introduction

Nowadays information about nearly every aspect of people’s lives can easily be accessed. Surprisingly one will soon reach the limits of information available regarding the components and conditions of manufacturing consumer products especially when it comes to mineral raw materials contained in products we use every day.

There is no information available about tantalum used in smartphones and whether it originates from the Democratic Republic of Congo, Australia, or Brazil, and whether tungsten, which is required for the vibration alarm in smartphones, was mined in China, Russia, or even in Austria. In the food sector, proof of origin methods and certifications systems are already in place. In the raw materials industry, proof of origin certifications are not properly established. However, certificates of origin for some raw materials (e.g. gold, gemstones, 3 T, and several others) are already being used on the market.

Consumers do not have the choice when buying products in which mineral raw materials are contained because they simply do not have any access to information, based on which they then make their decisions. Modern technology can be used to establish informed decision-making. With technical and technological possibilities nowadays, transparency and traceability can be provided in supply chains. Supply chains must be disclosed, and companies have to work backwards towards the point of provenance.

In addition, visibility into how materials and goods enter and move through the chain is on demand. People need to see what is happening everywhere in the supply chain and any material within should be traceable: From the mine to the processing plant, to the factory, to the customer, and beyond. Today, the technology definitely exists to turn that need into an operational reality. Information at the beginning of the supply chain needs to be verified and then traced along the whole supply chain. In the end that information needs to be shared with everyone involved. Raw materials are at the very beginning of the value chain and therefore they have a major impact on other business areas further down the supply chain. Society’s interest in the responsible extraction of raw materials is growing and legislative pressure is increasing.

## Transparency is no Longer Negotiable

At least since the collapse of many supply chains as a result of the COVID-19 crisis and the war in Ukraine, the interdependence of the world economy has reached the awareness of the broad public society. These two events shifted the focus to supply chain resilience. The systems had shown to be fragile and not resilient at all. Consumer trust, social license, and the freedom to operate are currently at risk. The COVID-19 crisis was followed by the war and resulted in supply bottlenecks for important raw materials in international trade. These include not only energy sources, precious metals, agricultural goods but also fertilizers and noble gases. Suddenly, everyone was interested in the origin of consumer goods, and the provenance of raw materials suddenly started to play an important role.

The European Union has imposed a number of import and export restrictions on Russia as part of the economic sanctions. Import restrictions include not only energy resources but also gold, steel, and iron as well as cement. Russia produces about 330 tons of gold per year, which makes it the second largest producer of gold worldwide [1]. Russian refineries were removed from the so-called “Good Deliveries List”. All of a sudden, the supply chain of gold and the origin of this raw material is of global importance and a hot topic. Of course, Russian mine gold continues to be sold to refineries in India and China, from where it is traded again as an unsuspicious commodity. Transparent supply chains are not always easy to implement, but the demand for them has never been higher.

In addition to that, the global production has changed dramatically in the last few decades. The production of cars, textiles, electronic goods, medicine, and even food was increasingly divided between different locations and organized in global production networks. A globalized world economy needs global rules for companies, and exactly this is the point where supply chain laws will intervene in the future. Resilient and sustainable supply chains offer a competitive advantage, but they require companies to have a clear visibility throughout the whole value chain and the ability to trace both inputs and outputs.

Companies have to be prepared for supply chain interruptions, they have to secure critical materials (minerals) input. Recent events showed that the global supply chains can be a limitation, yet, they can also be an opportunity for a company’s growth. Companies have to understand who and where they are sourcing from to ensure their future production. Additionally, they have to communicate their sustainability efforts to consumers in a trusted manner.

## Governments and Regulators are Already Acting

Society’s interest in the responsible extraction of raw materials is growing and legislative pressure is increasing. Companies have to be compliant with the upcoming regulations. The US Dodd-Frank Act, the European Conflict Minerals Regulation, the European Supply Chain Law or the UK’s Modern Slavery Act, just to name a few, are in full force and apply to supply chains in which mineral raw materials are involved.

The Conflict Minerals Regulation, which is based on the OECD Due Diligence Guideline, has a global scope and has addressed supply chain obligations since 2017 for importers of tin, tantalum, tungsten, and gold sourced from conflict-affected and high-risk areas. It is currently applied to the countries Democratic Republic of Congo (DRC), Colombia (Gold), Burkina Faso, Mali, and Niger [2].

The US Dodd-Frank Act has been legally binding since 2010 and also includes disclosure and reporting obligations for US listed companies regarding the use of certain raw materials (tantalum, tin, tungsten, and gold) that come from the Democratic Republic of Congo (DRC) or its neighbouring countries (Angola, Burundi, Rwanda, Zambia, Sudan, Tanzania) [3].

The European Supply Chain Act will come into full force in 2023, with the aim of obliging EU companies to contain or combat human rights violations and environmental destruction along their supply chain, no matter which raw material [4].

The UK Modern Slavery Act was put in place in 2015 to eliminate the risk of modern-day slavery in business operations and global supply chains. Companies must provide their annual report and its transparency in the supply chain clause [5].

These legal obligations are crucial. In the end, companies are unlikely to take action without the appropriate laws, and it is expected that several raw materials (especially critical raw materials) and other regions, such as conflict regions, are to be included in these regulations and laws. Furthermore, industry and company initiatives for certification systems for raw materials have been emerging within the last 20 years in order to address exactly these challenges. Environmental and social aspects in mining and the associated value chains have to be taken into account. Usually these systems relate to a certain raw material or a smaller group of minerals or metals, and they only relate to a certain part of the value chain. Therefore, further harmonisation is urgently needed. Standard systems that address all mineral resources are described below: The IRMA (Initiative for Responsible Mining Assurance), which is supposed to be a standard for responsible mining. However, it addresses only the part of mining and on-site processing in the upstream supply chain. IRMA is an industry-led initiative. The ICMM (International Council on Metals and Mining) developed from 2002 onwards and was one of the first sustainability standards with significant developments, but it only addresses the mining and on-site processing in the supply chain. Within the CERA4in1 (Certification of Raw Materials) initiative, a standardised certification scheme, containing four sub standards (4 standards in 1 system—4in1) for all raw materials will be developed. The market-ready CPS (CERA Performance Standard) is already advanced further and covers the whole upstream supply chain from the mine with the on-site processing to the processing and smelting outside the mine. Further standards within the system CERA 4in1 are planned to cover the whole supply chain, including exploration, intermediate products and end products. This affects all players, up- and downstream, from the mine to the customer, until the re-use and recycling of the product. It is still a long way to go but a huge progress is expected within the next years, as the CERA4in1 project would be a harmonization of most existing standards into one standard applicable to all raw materials. Other standard systems, such as the RJC (Responsible Jewellery Council), which includes the RJC Code of Practices and the RJC CoC (Chain of Custody Standard) cover the whole supply chain, they can, however, only be applied to certain raw materials, e.g. diamonds, gold, silver, and PGE. Another system for the whole supply chain is the ASI (Aluminium Stewardship Initiative), which, however, is only valid for aluminium (bauxite and alumina) [6]. There is a need for a harmonisation of the existing systems to enable clear certification and applicable standards for the raw materials industry.

## The Traceability of Raw Materials Along Their Supply Chains

According to ISO 2015b, traceability is the ability to trace the history, application, or location of an entity by means of recorded identification. To ensure the traceability of raw materials, there is a broad number of methods and tools available. By default, there are transaction documents in paper or electronic evidence. But how trustworthy is this type of traceability? Raw materials are extracted, processed, and are mixed with material from different origins, often including recycled materials. The situation is complex and the industry needs solutions away from conventional methods. There are systems that rely on an external tracer for physical labelling, such as the RFID technology, which is based on so-called tags, which are objects that have two components, one for radio communication and a memory used for data storage. Tracing of materials in the mining sector using RFID is feasible and enables real-time tracking [7]. Also barcodes and QR codes are commonly used, but they are anything but forgery-proof and are often misused.

The blockchain technology is another important digital tool to secure an archive of recorded transactions stored on a distributed ledger; however, it only allows the storage of data as a digital solution and is not based on physical product/material verification. The blockchain approach cannot verify the data input or physical processes within the supply chain. Therefore, what is entered in this database is what eventually will be delivered to a customer. Entries into this decentralized database could be subject to intentional or accidental entry of wrong information. Companies need to make sure to have enough, but mostly the right information available to empower sustainable supply chains with blockchain technology.

In the end, there is only one trustworthy method which is correlated to the raw material and its composition itself. The analytical proof of origin or analytical fingerprinting method is regarded as an appropriate term to describe analytical methods combined with data evaluation procedures developed to trace materials back to their source(s) [8]. It investigates the relationship between a raw material and its environment by analysing measurable and quantifiable material properties, such as major and trace elements, radiogenic and stable isotopes, accompanying mineralogical composition, grain properties, or crystallographic parameters. These parameters are naturally introduced into the raw material from the environment and genetic conditions, similar to human DNA. Information from materials of known provenance is used to create a reference database for a commodity. This “fingerprint” allows to verify a product back to a specific point of origin from ideally any point in the supply chain. Analytical proof of origin methods for raw materials are successfully applied under the following assumptions: (1) minerals have measurable compositions which differ depending on their genesis; (2) minerals from an area of enrichment (orebody) are more closely related to each other than to the same mineral from a second zone of enrichment, e.g. a different ore body [9].

Which method can finally be applied is very individual and depends on the respective raw material and the quantifiable parameters. Often a combination of different methods will be appropriate. In practice, fast, reliable, and low-cost methods are preferred over ultra-precise high-technology methods to ensure widespread use of the method and global comparability of data.

After having developed and tested analytical fingerprinting technologies for various ore types, such as zinc sulphide concentrates in the CERA project, members of the Chair of Geology and Economic Geology are currently working on method development for an analytical proof of origin for natural graphite. This will become the first analytical fingerprinting method developed for an industrial mineral. In the end, a digital database involving all relevant locations of natural graphite deposits worldwide will be developed. Graphite is an important industrial mineral commodity with a forecast significant increase in demand for batteries, especially in the electromobility sector. Major suppliers range from China, India, Mozambique, Sri Lanka, and Madagascar to Brazil, Russia, and Ukraine; European production is very limited. In the future, graphite samples traded to European industries could be analysed using appropriate methods to ensure their origin. With a machine learning algorithm, samples can be assigned to their “mother” sample with a high degree of certainty.

## The Future of Traceability

What is the purpose for establishing transparent and trustworthy supply chains from both the consumer’s and the producer’s perspective? Companies should value their suppliers. There are responsible companies at every stage of the value chain with which should be collaborated. They are working hard to ensure their workers are well paid under proper labour conditions and ensuring that they manage water, waste, and chemicals in the most responsible form. If companies know who their suppliers are and how they work, they can create more sustainable products in the end, enable informed decision-making, and communicate their sustainability efforts to consumers in a trusted manner. This allows them also to justify higher prices. Consumers put money into the value chain at the very end. Important aspects for them are where and to whom the money is going to and what this money is funding. Consumers are estimated to request more and more information in the future. They want to know where the products and materials come from and they care how they are being made. Consumer trust is central. Nevertheless, not only consumers are concerned but also media, employees, investors, and regulators start to care now, too. The origin of consumer goods has never been as relevant as it is now. Regardless of the form it takes, traceability will grow in impact, credibility, and cost-effectiveness. More and more companies will develop their own initiatives or join existing ones as a fundamental part of their supply chain management. Privacy protection in the certification systems must still be provided and ensured.
